# Application of Reinforcement Learning in Multiagent Intelligent Decision-Making

**DOI:** 10.1155/2022/8683616

**Published:** 2022-09-16

**Authors:** Xiaoyu Han

**Affiliations:** Hunan University, Juzizhou Street, Yuelu, Changsha, Hunan, China

## Abstract

The combination of deep neural networks and reinforcement learning had received more and more attention in recent years, and the attention of reinforcement learning of single agent was slowly getting transferred to multiagent. Regret minimization was a new concept in the theory of gaming. In some game issues that Nash equilibrium was not the optimal solution, the regret minimization had better performance. Herein, we introduce the regret minimization into multiagent reinforcement learning and propose a multiagent regret minimum algorithm. This chapter first introduces the Nash Q-learning algorithm and uses the overall framework of Nash Q-learning to minimize regrets into the multiagent reinforcement learning and then verify the effectiveness of the algorithm in the experiment.

## 1. Introduction

Strengthening learning allows agent to continuously undergo self-learning through the way of interacting with the environment and ultimately meets the expected goals. It is a type of try and error (TE) learning. TE learning first appeared in the study of cats in 1898, and he conducted TE experiments on cats [1]. Watkins and Dayan proposed the famous Q-learning algorithm in 1989 [2], which combines time series difference with the Markov decision process (MDP) and Bellman equation. It is the most classic algorithm of reinforcement learning. In 2015, Mnih proposed the deep Q-learning network [3] (DQN) by combining the deep neural network and Q-learning and achieved superhuman performance in the game. Since then, reinforcement learning has fully entered the deep reinforcement learning stage.

The achievements of deep strengthening learning are remarkable. DQN could achieve better results in 49 ATARI games than professional players. Google's DeepMind team created the Go robot AlphaGo in 2016, which uses the DQN network and could learn itself. AlphaGo won the game with the top Go master of human beings, and Li Shishi's games won all of them [4]. In-depth reinforcement learning has also been applied to solve problems such as MUJUCO [5] and 3D maze. In-depth strengthening learning has become the most potential research direction in the complexity of the real world and has also made great contributions in the field of artificial agent.

It is difficult to migrate to multiagent in the success of a single agent. Multiagent reinforcement learning (MARL) and single agent reinforcement learning from the state of the most different one is determined by multiple agents rather than a single agent. As the environment becomes uncertain, each agent must face the optimal strategy movement problem; that is, the optimal strategy is constantly changing with the changes in other agent parties [6, 7]. Because of this, most of the single agent reinforcement learning algorithms are not effective in the multiagent environment, and as the state action space of each agent grows in the index level, the problem of Gaowei curse have become more serious in the multiagent environment. Hence, MARL proposes a series of new technologies and methods to achieve the purpose of accelerating the entire learning process through sharing of knowledge and learning between agent [8, 9].

Another difference between multiagent and single agent to enhance learning is that there are multiple agents, and there could be cooperation or competition (game) relationships between agents, and this relationship is determined by the reward function [10]. If there is a purely cooperative relationship between the agents, their reward functions are the same, and the learning goals are to maximize the common income. If there is a purely competitive relationship between the agents, their reward function is zero-sum. If there is neither a complete cooperation nor a complete competition, their relationship is called mixed cooperation and competition [11]. Main challenges of multiagent reinforcement learning are the instability of the environment, some observations, rewards allocation, and computing complexity. The calculation complexity is a problem that all multiagent learning algorithms have to face. The calculation complexity contains other challenges.

The combination of regrets in online learning and the combination of multiagent strengthening learning is a new direction in the development of multiagent in recent years. Online learning is a type of machine learning. Unfortunately, the difference between the rewards related to the action and the return he actual actions is big [12], the regret minimum related algorithm in the online learning could be used to solve the problem of expansion game under nonperfect information, such as the virtual regret minimization (Counterfactual Regret Minimization, CFR [13]) and its latest variant CFR+ [14]. CFR could be achieved in the game of double zero and nonperfect information game to Nash balance. Brown and Sandholm could defeat top professional players in one-to-one Texas Poker with the help of Sandholm. Noam Brown's agent Pluribus [15] could achieve the same excellent results in the six Texas Poker. Noam Brown and others combined CFR with deep neural networks to propose a deeper virtual regret minimum algorithm (DCFR [16]). In the field of multiagent reinforcement learning, the deep role [17] algorithm proposed by combining CFR and self-play could identify partners and opponents in the game. A regret-based minimization algorithm (ARM [18]) was proposed by Jin et al in order to combine the concept of advantageous functions in enhanced learning with CFR. Steinber [19] also combines CFR and enhanced learning. All these items should be redirected to MARL based on the Nash Q-learning framework.

Nash Q-learning [20] use Nash balance to solve the problem of zero-sum game under the multiagent body. NASH Q-learning is an early work of multiagents to strengthen learning. In the face of the instability of multiagent reinforcement learning, it is not possible to use neural networks for powerful search, but to search each agent first, whenever, their decisions are based on Nash balance. The author also proves the convergence of the algorithm under the theoretical level. This article draws on this idea to give the agent a priori knowledge so that their decisions are minimized based on regret and proposes multiagent regret minimum algorithms. Multiagent regrets the minimum algorithm compared to Nash Q-learning, which could not only deal with zero-sum game problems but also handle nonzero-sum games. In Nash Q-learning, the *Q* function has made two changes in two aspects. The first is to overcome the instability. Another is the input of the *Q* function of the current state and the joint action.

We herein propose a multiagent regret minimization and strengthen the learning (MARMQ) algorithm. MARMQ was above the Nash Q-learning framework. The selection part of the counterpart was improved and Lemke–Howson algorithm was replaced with iterative regret minimum algorithms not looking for the Nash balance point but to minimize the motion of each agent. At the same time, when the *Q* value was updated, the *Q* value of the minimum movement in the next state was used as part of the update and ultimately enabled the agent to learn the regret minimum strategy.

## 2. Algorithm Design and Analysis

### 2.1. Construct of Multiagent Regret Minimization Q-Learning (MARMQ)

Changing the income function U to the *Q* function, it was easy to introduce the relevant concepts in iterative regret minimization algorithms into multiagent strengthening learning.

**Definition 1 .**

DO was defined as delete operator, DO (*Q*_*i*_, *A*_*i*_) deleted the movement of the agent *i* in action space *A*_i_ according to *Q*_*i*_ and did not have the minimum regret value to delete it, and only retained the action with the minimum regret:

(1) $$\begin{array}{c} DO\left(Q_{i},A_{i}\right)=\left\{a_{i}:\underset{a_{i}\in A_{i}}{Regret}\left(a_{i}\right)=Regret\left(A_{i}\right)\right\}\text{.} \end{array}$$

**Definition 2 .**

The DO_total_ operator was defined as not deleting the mobility of the joint action space of the agent:

(2) $$\begin{array}{c} {DO}_{total}\left(Q_{1},\ldots ,Q_{n}{,A}_{1},\ldots ,A_{n}\right.=DO\left(Q_{1},A_{1}\right)\times \cdots \times DO\left(Q_{n},A_{n}\right)\text{.} \end{array}$$

After deleting a movement space to minimize the nonregrets, the remaining movement constituted a new action space. The regret of the action of this new action space may change, and it was necessary to delete the nonregret minimization of the movement until the action space no longer changes.

**Definition 3 .**

The *DO*_total_^*∞*^ operator was to minimize the computing son to iterate. It would repeat deletion unless regret minimum combined action until the joint action space no longer changed:

(3) $$\begin{array}{c} {DO}_{total}^{\infty }\left(Q_{1},\ldots ,Q_{n}{,A}_{1},\ldots ,A_{n}\right)={DO}_{total}^{i}\left(Q_{1},\ldots ,Q_{n}{,RM}_{total}^{i-1}\right)\text{,}\\ {DO}_{total}^{i}={DO}_{total}^{i-1}\text{.} \end{array}$$

When the action space of each agent was limited, *DO*_*total*_^*∞*^ must exist because the deletion of action could not be continued. Therefore, in order to ensure the feasibility of the algorithm, MARMQ was only suitable for the environment with limited space and could not solve the problem of continuous action space.

To execute the calculation and update the *Q* value, we need to redefine the *V* function and *Q* function under multiagent to strengthen learning.

**Definition 4 .**

In random games, the *v* function of agent *i* was defined on (*s*, *b*_1_,…, *b*_*n*_), which was the expectation of future return when all agent parties execute the regret minimum strategy. AKI means the intelligent agent which is the action space of the body I at *k* times:

(4) $$\begin{array}{c} v_{i}\left(s,b_{1},\ldots ,b_{n}\right)=v^{i}\left(s,{DO}_{total}^{\infty }\right)=E\left\{\overset{\infty }{\underset{k=0}{\sum }}c^{k}r_{i,k+1}\left|\left.s_{0},{DO}_{total}^{\infty }{\left(Q\right.}_{1}^{k},\ldots ,Q_{n}^{k}{,A}_{1}^{k},\ldots ,A_{n}^{k}\right)\right.\right\}\text{.} \end{array}$$

**Definition 5 .**

The *Q* function of agent *i* was defined on (*s*, *d*^1^,…, *d*^*n*^), which was the reward of the current joint action and the expectations of all agent parties when the regret minimum strategy was executed:

(5) $$\begin{array}{c} Q_{i}\left(s,d_{1},\ldots ,d_{n}\right)=r_{i}\left(s,d_{1},\ldots ,d_{n}\right)+e\underset{s^{\prime }\in S}{\sum }p\left(s^{\prime }\left|s,d_{1},\ldots ,d_{n}\right.\right)v_{i}\left(s^{\prime },b_{1},\ldots ,b_{n}\right)\text{.} \end{array}$$

The updated formula of *Q* value is

(6) $$\begin{array}{c} Q_{i}^{t+1}\left(s,d_{1},\ldots ,d_{n}\right)=\left(1-f_{t}\right)Q_{i}^{t}\left(s,d_{1},\ldots ,d_{n}\right)+f_{t}\left[r_{i}^{t}+eQ_{i}^{t}\left(s^{\prime },b_{1}\left(s^{\prime }\right),\ldots ,b_{n}\left(s^{\prime }\right)\right)\right]\text{.} \end{array}$$

Formulas (5) and (6) were regret minimization strategies.

(7) $$\begin{array}{c} b_{1},\ldots ,b_{n}=DO_{total}^{\infty }\text{.} \end{array}$$

Although MARMQ and Nash Q-learning had many similarities, there is a difference about how to use the Q value of the next state to update the value of the current state. Nash Q-learning calculated a balanced strategy with the *Q* table of all agents in the following state and used the internal accumulation of a balanced strategy and the vector of the *Q* value of each agent as part of the *Q* value of the update. The *Q* values of all movements should be iterative regret minimization calculations to find the minimum movement of regret and use the *Q* value of the action as part of the update. In contrast, the single agent was used to directly select the optimal *Q* value. The specific differences are shown in Table 1.

### 2.2. Traveler Game under MARMQ

First, the performance of the MARMQ algorithm would be verified in the traveler's game. The common characteristics of traveler game and centipede game were that agent parties could choose to cooperate to increase overall returns, but at the same time, competition could also be selected to reduce the benefits of the other party. The higher the degree of cooperation, the greater the income; the lower the degree of cooperation, the smaller the income. There were two advantages while fighting was two defeats.

There were two players in the traveler's game, which were quoted between 2 and 100, respectively. The income of the players with high offer was reduced to the price of players with low quotation. Under the existing rules, if the two players choose to cooperate to increase their quotation, their income would increase. If players did not cooperate with each other, they only chose to maximize their own interests. Because people with low quotations had the highest benefits, they would choose to reduce their quotation, but everyone's income would be reduced.

**Figure 1:**
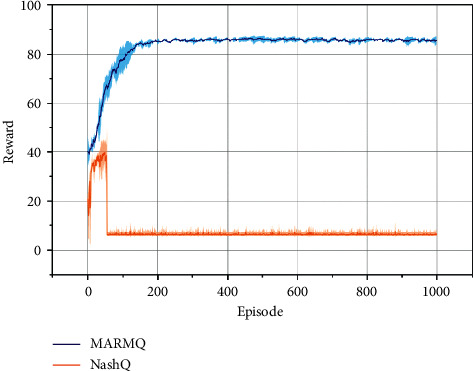
Experimental results of the traveler game.

The Nash balance solution was that both players would choose the minimum quotation 2, and the tolerance of iterative regret was 97. The experimental results are shown in Figure 1. The benefit of players in MARMQ was about 88, and Nash Q-learning was about 8. The income was 88 that the lowest quotation of the two players was 86, which was much better than Nash Q-learning. Regret minimization under the player's game in the game of travelers tend to cooperate to raise quotes, and MARMQ uses regret minimization strategies to choose behaviors and updates *Q* to make players tend to increase the quotation so that MARMQ could get higher returns. In this scenario, Nash balance was the worst solution, so Nash Q-learning used Nash equilibrium as a strategy of agent. Each agent was also in line with expectations that the effect of low quotation was also in line with expectations.

### 2.3. Centipede Game under MARMQ

**Figure 2:**
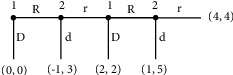
Flow diagram of the centipede game.

The centipede game is shown in Figure 2. In the centipede game, players 1 in the first round first make decisions. If you chose not to cooperate (D), the game would be over directly. Player 1 gets 0, and player 2 gets 0. If the player 1 chose (R), it would enter the second round, and it was the turn of the player 2 to choose. If player 2 chooses not to cooperate (D), the game would be over directly. Player 1 gets 1, and player 2 gets 3. If player 2 chooses cooperation (R), it would give the right to the opponent and then enter the third round of the game. In the third round, if the player 1 chooses not to cooperate (D), the game would be over directly, player 1 gets 2, and player 2 gets 2. If the player 1 chooses the cooperation (R), it would enter the fourth round and the player 2 gets to choose. If player 2 chooses not to cooperate (D), the game would be over directly. Player 1 gets 1, and player 2 gets 5. If the cooperation (*R*) was selected, the game would be over, and both sides would get 4.

First of all, to calculate the solution of the next iteration of the regret minimum algorithm on the game, the first round of regret matrix calculated based on the income, as shown in Table 2. For convenience, player 1 in the matrix could choose betrayal in the first, third, and fifth rounds, but in the fourth round, the actual game ends. So the fifth round of betrayal of player 1 represents that he chose cooperation in the third round. Player 2 choosing the sixth round of betrayal represents cooperation in the fourth round. According to calculations, players' regrets of betrayal in first, third, and fifth rounds were 4, 2, and 1, respectively, so player 1 would delete the first and third round betrayal strategies. Players' regrets in the second, fourth, and sixth rounds of betrayal were 2, 1, and 1, respectively, so Player 2 would delete the second round of betrayal strategies and then entered the second round of strategy to delete.

The second round of the regret matrix is shown in Table 3. One player had one strategy, so there was no need to be deleted, and the regrets of the player 2 in the 4th and 6th rounds of betrayal were 0 and 1, respectively. So under the iterative regret minimum algorithm, player 1 would choose to cooperate until the end, and player 2 would choose to betray in the fourth round.

**Figure 3:**
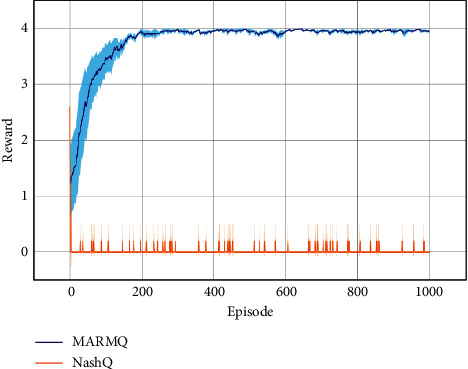
Experimental results of the centipede game.

The experimental results are shown in Figure 3. The benefits of the two players in Nash Q-learning were about 0, indicating that the strategy of the first player was to choose to betray the first round, which was consistent with the balance of Nash. Under the Nash balance strategy, player 1 did not allow the game to enter the second round in order to maximize their own interests because players who also executed the Nash balance strategy 2 would choose to end the game in the second round and make themselves. To maximize interests, the benefits of player 1 in this time were less than that in the first round of betrayal.

The results of Nash Q-learning were related to each agent in the algorithm. MARMQ's income was 4, which proves that all players had chosen to cooperate to the end. Based on the previous analysis, unfortunately the minimization strategy would make players to give up in the fourth round under a single cricket game. Player 1 benefits 1, and player 2 benefits 5. However, when the game was repeated, the player's strategy had changed, and they would choose to cooperate till the end. It is because under the repeated game, each player could choose to punish the other party's unsuccessful behavior. At this time, the value was far less than the betrayal, so the final result of the two parties moved to cooperation.

### 2.4. Grid-World under MARMQ

**Figure 4:**
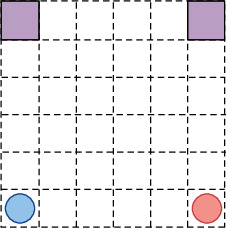
Game rules of grid-world.

Grid-World was a classic experimental environment for enhanced learning. Papers such as Nash Q-learning were used to test the performance of the algorithm. There were two agent parties in Grid-Worm. As shown in Figure 4, each agent started in the corner of the grid. The target of each agent was to reach any target position at the top. The agent could only move one grid in each step, and the direction of the movement could only be four directions in the upper and lower left and right and could not move obliquely. If the two agent parties tried to enter the same grid at the same time, a collision occurs, and each agent would not move. At the same time, it was punished by 10. In the end, the two agent parties were rewarded by 100; otherwise, each step was to be punished by 1.

Under the above rules, for the purpose of maximizing income, MARMQ must ensure that both could go to the target position as soon as possible. Because there were two target positions, there was no competitive relationship between the agents; otherwise, they need to cooperate with each other to avoid collisions and reach the target position as soon as possible. At the same time, the goals of the two agent parties could not be the same. If both agent parties chose the left target, then the game would not end. It could move towards your recent goals. It was difficult for the agent parties to learn independently to complete different goals while not colliding.

**Figure 5:**
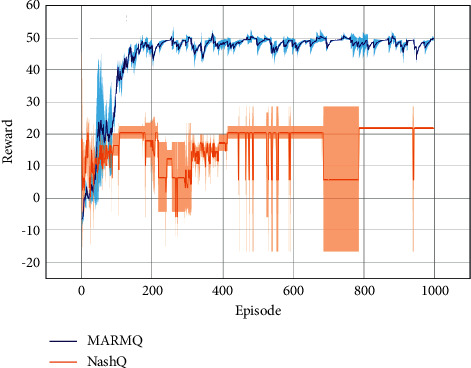
Experimental results of Grid-World.

Set the size of the map to 3 × 3 in the experiment in this article. The results of the experiment are shown in Figure 5, where the *y*-axis represents the total income of the agent at the end of this round, except for the number of steps used, which meant the average return of one-step. MARMQ's final single-step reward was about 50, and Nash Q-learning was only about 20, indicating that MARMQ was shorter than Nash Q-learning. Both algorithms could make the agent reach the target position, but the number of steps used by the two agent parties in MARMQ was less than Nash Q-learning. In Nash Q-learning, the two agents did not choose the optimal path to reach the target, or the agent chose a relatively far away position to make the goal, so that the single-step income of the two agent parties was reduced. The differences between the results of MARMQ and Nash Q-learning showed the different strategies of Nash equilibrium and regret minimalization strategy. The regret minimization strategy tended to cooperate and considered the maximum of global interests. The reaction on the algorithm Nash Q-learning would only make the agent reach the target and would not make the overall benefits the greatest, and MARMQ would consider global returns. Another advantage of MARMQ was that MARMQ showed low deviation and high reproducibility while the error band of Nash Q-learning was larger and very unstable.

## 3. Conclusion

In summary, we had used regret minimization the study of multiagent and put forward the MARMQ algorithm, and compared it with Nash Q-learning. The results showed that MARMQ's performance in Travelers Game, Centipede Game, and Grid-World was better than Nash Q-learning. It showed that when MARMQ uses regret minimization strategy of action, it was better than the Nash strategy that was more conducive to the maximization of its own interests. Nash Q-learning was the maximization of their own interests as the starting point. This would often fall into the predicament of the prisoner, and MARMQ was seeking to minimize the regret, which relieved this trap to a certain extent. We also proposed another form of training in MARMQ, i.e., independent training. In independent training, each agent could no longer use the *Q* table of other agent to minimize regret but to learn the *Q* table of each agent. Such independent training had undoubtedly increased time costs, but the performance in the traveler's game was very good. All algorithms and trainings could pave the way to the direction of future research.

## Figures and Tables

**Table 1:** Definition of Q value under different algorithms.

	Single	Nash	Regret
*Q*	*Q*(*s*, *a*)	*Q*(*s*, *d*_1_,…, *d*_*n*_)	*Q*(*s*, *d*_1_,…, *d*_*n*_)
Updated *Q*	Largest value under the next state	Product of agent's united Nash strategy and *Q* value	*Q* value of minimum action under next state's regret value

**Table 2:** Regret matrix of the first round.

Player 2				
Player 1		2 (2)	4 (1)	6 (1)
	1 (4)	0,0	2,0	4,0
	3 (2)	1,0	0,1	2,1
	5 (1)	1,2	1,0	0,1

**Table 3:** Regret matrix of the second round.

Player 2			
Player 1		4 (0)	6 (1)
	5 (0)	0,0	0,1

## Data Availability

The dataset can be accessed upon request.
